# Disentangling Anhedonia and Self-Stigma in Schizophrenia Using Bayesian Network Analysis

**DOI:** 10.31083/AP50731

**Published:** 2026-07-28

**Authors:** Jiabao Chai, Dayun Jiang, Hanxue Yang, Lin Liu, Fuquan Liu, Weigang Pan, Xiaohong Li

**Affiliations:** ^1^Peking University Huilongguan Clinical Medical School, Beijing Huilongguan Hospital, Capital Medical University, 100096 Beijing, China; ^2^Cognitive Science and Allied Health School, Institute of Life and Health Sciences, Key Laboratory of Language and Cognitive Science (Ministry of Education), Beijing Language and Culture University, 100083 Beijing, China

**Keywords:** schizophrenia, anhedonia, self-stigma, social support, life quality

## Abstract

**Background:**

The interrelationships among the negative symptoms of schizophrenia and factors affecting quality of life remain unclear. To address this gap, the present study explores the complex interrelationships among anhedonia, self-stigma, self-esteem, psychological resilience, and social support in individuals with schizophrenia.

**Methods:**

A comprehensive battery of measures was used to assess 447 patients with schizophrenia. Undirected network analysis was employed to examine the centrality of various factors, and Bayesian network analysis was used to explore the indirect influence of upstream variables on anhedonia through psychological factors.

**Results:**

Self-stigma was identified as the most central factor. Among the subdimensions of anhedonia, abstract anticipatory pleasure and contextual consummatory pleasure demonstrated the highest centrality. Negative symptoms of schizophrenia and social support were found to indirectly influence different aspects of anhedonia through psychological factors such as self-esteem and resilience. In one of the identified pathways, social support influenced psychological resilience, which in turn affected abstract anticipatory pleasure, contextual consummatory pleasure, and contextual anticipatory pleasure.

**Conclusions:**

Our findings suggest that social support is associated with pleasure experiences partly through psychological resilience and underscore the potential mediating role of psychological factors in linking clinical symptoms, social resources, and hedonic capacity in schizophrenia.

## Main Points

1. Self-stigma occupied the most central position in the overall psychosocial network of schizophrenia.

2. Among anhedonia-related subdimensions, abstract anticipatory pleasure and contextual consummatory pleasure showed the most prominent network centrality.

3. Bayesian network analysis revealed directional pathways linking social support and negative symptoms to hedonic functioning through intermediate psychological factors, particularly resilience and self-esteem.

4. These findings highlight self-stigma, self-esteem, resilience, and anhedonia-related domains as priority candidates for future assessment and intervention research in schizophrenia.

## 1. Introduction

Schizophrenia has traditionally been conceptualized within a psychopathological framework characterized by disturbances in affective responsiveness, associative functioning, and engagement with reality. Within this historical framework, negative symptoms have been understood as reductions in or absences of normal psychological functions. Contemporary research has revisited and refined this conceptualization within modern diagnostic and psychopathological perspectives [1,2].

Importantly, negative symptoms should not be interpreted as a unitary construct. Current literature distinguishes primary negative symptoms, which are considered intrinsic to schizophrenia, from secondary or pseudo-negative symptoms related to persistent positive symptoms, depressive symptoms, medication-related extrapyramidal or sedative adverse effects, substance use, or prolonged social and environmental deprivation [3,4]. In addition, cognitive and sociocognitive deficits may phenotypically overlap with avolition, alogia, and asociality, which complicates clinical interpretation [5]. This distinction is clinically and methodologically important because secondary negative symptoms may lessen when their underlying causes are identified and treated, whereas primary and enduring negative symptoms are often more treatment-resistant and may reflect a different therapeutic target [6]. Accordingly, reduced motivation, diminished activity, or social withdrawal should not automatically be interpreted as core negative symptom pathology without a careful consideration of alternative explanations.

Negative symptoms play a key role in diminishing pleasure experiences. Individuals with schizophrenia reported substantially lower scores on abstract anticipatory pleasure (i.e., the enjoyment anticipated from future events that are abstract or symbolic rather than from specific present situations [7]) and contextual consummatory pleasure (the enjoyment felt while directly interacting with specific situational stimuli [7]) compared with healthy controls. Greater severity of negative symptoms was found to be associated with reductions in anticipatory pleasure, although the same relationship was not consistently observed for consummatory pleasure [8,9]. Furthermore, impairments in anticipatory (rather than consummatory) pleasure are known to be particularly pronounced in individuals with schizophrenia and strongly linked to community involvement and participation in daily activities [10,11]. Gender differences were observed in Temporal Experience of Pleasure Scale (TEPS) scores, with females and males reporting higher anticipatory and consummatory pleasures, respectively [12]. Similarly, gender differences were observed in Positive and Negative Syndrome Scale (PANSS) scores, with males and females showing more pronounced positive and negative symptoms, respectively [13].

Another factor associated with negative symptoms is self-stigma. Self-stigma refers to the process by which individuals with mental disorders internalize societal stereotypes and prejudices toward mental illness and consequently develop negative self-views [14]. In line with Corrigan’s cognitive-behavioral framework of self-stigma, the internalization of societal stigma may erode self-esteem and diminish motivation to pursue meaningful life goals [15]. Numerous studies have highlighted self-stigma as a substantial impediment to recovery, particularly in individuals with schizophrenia, where it obstructs adherence to treatment and hinders participation in rehabilitation programs [16]. Moreover, self-stigma may adversely affect the emotional well-being and psychological adjustment of individuals with schizophrenia, curbing their ability to engage with social support networks [17,18]. Individuals with high levels of self-stigma have been shown to exhibit avoidance behaviors in social interactions and experience notable declines in work performance during recovery phases [19,20].

Meta-analytic and cross-sectional studies suggest that self-stigma is associated with negative symptoms of schizophrenia, poorer functional outcomes, and reduced social involvement [21,22]. Higher internalized stigma, lower self-esteem, and diminished social support negatively impact life quality, with self-esteem mediating the stigma–life satisfaction relationship and social support moderating psychosocial outcomes [11]. Deficits in anticipatory pleasure are particularly evident in schizophrenia and associated with key functional outcomes [10,11]. These findings illustrate the complex interplay between self-stigma, self-esteem, social support, and pleasure, all of which significantly influence life quality and functional outcomes in individuals with schizophrenia. However, efforts into finding intervention targets are still required, partly because of the lack of frameworks elucidating the complex relationships among the abovementioned factors.

Network models of psychopathology have attracted considerable attention as a framework for capturing the complex relationships among the heterogeneous dimensions of mental disorders [23]. Within such models, constructs are represented as nodes, and connections between them are depicted as edges [24]. Unlike traditional linear statistical approaches, which often simplify associations by assuming independence or linearity, network approaches conceptualize psychiatric disorders as dynamic systems of interrelated psychological variables, with direct and indirect relationships collectively defining the network structure [25]. This framework facilitates the identification of central nodes (variables strongly influencing the network) and critical pathways that may represent promising targets for intervention [25]. Bayesian network analysis is a complementary technique offering a principled approach for investigating potential directional relationships among multiple disorder-related nodes and addressing the limitation of nondirectionality inherent in traditional network models [26]. Such approaches are increasingly used to elucidate how specific symptom dimensions may influence other symptoms and thus identify pathways through which symptom clusters exacerbate overall severity [26,27]. Thus, we herein utilized undirected and Bayesian network analyses to examine the structural relationships among psychological symptoms and psychosocial factors in a large clinically diagnosed schizophrenia sample.

Based on previous evidence, we hypothesized that (1) in undirected network analyses, self-stigma or anhedonia-related variables would exhibit the highest centrality; (2) self-stigma, self-esteem, negative symptoms, anhedonia, and social support would show closely related edges in an undirected network; and (3) in a Bayesian network, self-stigma may be negatively and indirectly associated with anhedonia-related subdimensions through self-esteem and other intermediate psychosocial variables. Gender differences were examined as exploratory descriptive analyses rather than as a core study hypothesis.

## 2. Materials and Methods

### 2.1 Participants

This study was conducted at Beijing Huilongguan Hospital between May and October 2023. During this period, 510 inpatients with clinically diagnosed schizophrenia were screened for eligibility. Of these, 52 were excluded based on the exclusion criteria listed below, and 11 more were excluded because of incomplete questionnaires. Ultimately, 447 participants were included in the final analysis.

All participants met the diagnostic criteria for schizophrenia according to the International Classification of Diseases (10th Revision, 1993) of the World Health Organization [28], and diagnoses were independently confirmed by at least two experienced psychiatrists. Diagnostic ascertainment was based on routine inpatient psychiatric evaluation, including medical history reviews, psychiatric interviews, physical examinations, and reviews of available medical records. Clinically indicated ancillary examinations were considered as part of usual care when needed; however, we did not uniformly implement a standardized research-specific battery of neuroimaging, electroencephalography, lumbar puncture, toxicology screening, or comprehensive laboratory testing across all participants. Exclusion decisions were therefore based on documented clinical diagnoses and medical conditions in the inpatient record, as judged by the treating psychiatrist/physician. The final sample comprised 232 males (52%) and 215 females (48%). The mean age was 49.09 ± 13.36 years, with males being significantly younger than females (47.82 ± 13.75 vs. 50.47 ± 12.81 years; *p* = 0.036). Written informed consent was obtained from all participants, and demographic and clinical information (including illness duration, current antipsychotic dose equivalent, medication exposure duration, length of current hospitalization, and the PANSS total score) was collected.

The inclusion criteria were (1) a diagnosis of schizophrenia according to the International Classification of Diseases (10th Revision); (2) stable antipsychotic treatment for at least 1 month prior to enrollment, defined as no change in the antipsychotic regimen (i.e., no medication switch or clinically significant dose adjustment) during this period; (3) an age of 18–65 years; and (4) at least a junior high school education to ensure adequate literacy and comprehension for completing the self-report questionnaire battery.

The exclusion criteria were (1) major physical illnesses that could interfere with assessment or study participation, including severe or medically unstable somatic conditions such as decompensated diabetes, acute cardiovascular events, or other treatment-refractory medical conditions; (2) brain disorders, including traumatic brain injury, epilepsy, dementia, or other neurologic conditions judged to substantially affect cognition or psychiatric presentation; and (3) intellectual disability, defined as clinically documented intellectual functioning equivalent to IQ <70 or recorded in the medical chart by a psychiatrist. Eligibility decisions were made by the treating psychiatrist/physician on the basis of routine clinical assessment and documented medical information in the inpatient record.

### 2.2 Psychopathological Assessment

Symptom severity was rated independently by 3 psychiatrists following joint calibration with PANSS scores as anchor vignettes. Raters met regularly during data collection to monitor potential drift. Interrater reliability was evaluated in a subset of interviews rated in parallel (*n* = 3) using two-way random-effects, single-measure intraclass correlation coefficients (ICCs) (2,1). These parallel ratings were part of a pragmatic calibration procedure during routine clinical data collection rather than a formal reliability substudy, and only a limited number of jointly rated interviews were feasible in the inpatient setting. The ICCs were 0.84 for the PANSS negative scale (95% confidence interval (CI): 0.76–0.90), 0.82 for the positive scale (CI: 0.73–0.89), 0.80 for the general psychopathology scale (CI: 0.70–0.88), and 0.92 for the PANSS total score (CI: 0.88–0.95), indicating good to excellent agreement. Given the small number of calibration interviews, reliability estimates were interpreted cautiously. Rating consistency was further supported by routine cross-ratings, case conference reviews, and adjudication procedures. The employed scales are listed below.

#### 2.2.1 PANSS

PANSS scores were used to assess positive symptoms (PANSS-P), negative symptoms (PANSS-N), and general psychopathology. Items were rated on a 7-point scale from 1 (absent) to 7 (extreme), with the total score ranging from 30 to 210. Higher scores indicated greater symptom severity. The internal consistency of the PANSS was previously reported as acceptable to good (Cronbach’s *α* >0.70) [29] and found to be acceptable in the present sample (Cronbach’s *α* = 0.85).

#### 2.2.2 Self-Esteem Inventory (SEI)

The SEI consists of 58 items rated using a dichotomous response format (1 = “like me”. 0 = “not like me”), including 30 reverse-scored items. Total scores range from 0 to 50, with higher scores indicating higher levels of self-esteem. The SEI demonstrated good internal consistency previously (Cronbach’s *α* = 0.86) [30] and in the present sample (Cronbach’s *α* = 0.82).

#### 2.2.3 Connor–Davidson Resilience Scale (CD-RISC)

Resilience was assessed using the CD-RISC, a 25-item measure scored on a 5-point Likert-type scale from 0 (“not true at all”) to 4 (“true almost all of the time”). Total scores range from 0 to 100, with higher scores reflecting greater resilience. This scale showed high internal consistency previously (Cronbach’s *α* = 0.92) [31] and in the present sample (Cronbach’s *α* = 0.87).

#### 2.2.4 Internalized Stigma of Mental Illness (ISMI) Scale

Internalized stigma was measured using the ISMI scale, which comprises 29 items across 5 domains: alienation, stereotype endorsement, perceived discrimination, social withdrawal, and stigma resistance. Items are rated on a 4-point scale to afford total scores of 29–116, with higher scores indicating greater levels of internalized stigma. This scale showed excellent internal consistency previously (Cronbach’s *α* = 0.94) [32] and in the present sample (Cronbach’s *α* = 0.90).

#### 2.2.5 Schizophrenia Quality of Life Scale (SQLS)

This instrument includes 30 items organized into 3 domains, namely psychosocial functioning, motivation and energy, and symptoms/side effects. Items are rated on a 5-point scale (0–4), and domain scores are transformed to a 0–100 range, with higher scores indicating poorer life quality. The SQLS demonstrated acceptable internal consistency previously (Cronbach’s *α* = 0.72–0.83) [33] and in the present sample (Cronbach’s *α* = 0.84).

#### 2.2.6 Coping Questionnaire for Schizophrenic Patients (CQSP)

The CQSP comprises 54 items covering 4 domains: problem solving, avoidance, cognitive adjustment, and emotion regulation. Items are rated on a 5-point Likert scale from 1 (“never use”) to 5 (“always use”), with higher scores reflecting a more frequent use of the respective coping strategy. Subscale scores are calculated by summing item responses. This tool showed good internal consistency previously (Cronbach’s *α* = 0.68–0.95) [34] and in the present sample (Cronbach’s *α* = 0.78).

#### 2.2.7 Social Support Rating Scale (SSRS)

Perceived social support was measured using the SSRS, which includes 10 items assessing 3 components: objective support, subjective support, and support utilization. Total scores are obtained by summing all item scores, with higher scores indicating greater levels of social support. Based on established cutoffs, total scores of ≤22, 23–44, and 45–66 indicate low, moderate, and high support, respectively. The SSRS demonstrated acceptable internal consistency previously (Cronbach’s *α* ≈ 0.80) [35].

#### 2.2.8 TEPS

Hedonic capacity was assessed using the TEPS, which consists of 20 items assessing anticipatory and consummatory pleasure across abstract and contextual domains, yielding 4 subscales (abstract anticipatory, contextual anticipatory, abstract consummatory, and contextual consummatory). Items are rated on a 6-point Likert scale, with higher scores indicating a greater capacity to experience pleasure over time. The TEPS has been widely applied in schizophrenia research to evaluate motivational and hedonic processes and has shown good internal consistency and construct validity in clinical and nonclinical samples [36]. The Chinese TEPS showed good reliability (Cronbach’s *α* = 0.83) and stability (*r* = 0.79–0.81) previously [7] and exhibited good internal consistency in the present sample (Cronbach’s *α* = 0.84).

### 2.3 Grouping by Negative-Symptom Severity

To examine the relationships among negative symptoms, self-stigma, and life quality, participants were classified into high- and low-negative-symptom groups based on PANSS-N scores. A cutoff of 19 was applied, with scores of >19 indicating higher levels of negative symptoms and scores of ≤19 indicating lower levels. Although a PANSS-N score of 24 is commonly used to denote moderate negative symptom severity [37], a lower threshold was selected to capture earlier functional differentiation before the onset of moderate-level symptoms. Similar stratification strategies have been used to investigate psychosocial variability in subthreshold populations [38].

### 2.4 Statistical Analysis

Statistical analysis was carried out using the Statistical Package for the Social Sciences (SPSS 22.0, International Business Machines Corporation, Armonk, NY, USA), with reporting guided by the Strengthening the Reporting of Observational Studies in Epidemiology statement for observational designs [39]. Following data quality control and eligibility screening, 447 individuals comprised the analytic sample. Gender-related differences were assessed as part of descriptive analyses. Continuous measures were presented as means with standard deviations or medians with interquartile ranges when distributions were nonnormal, whereas categorical variables were reported as absolute frequencies and proportions. All network analyses were conducted using R version 4.5.2 (R Foundation for Statistical Computing, Vienna, Austria), available at https://cran.r-project.org/.

Undirected regularized network models were used to estimate conditional associations among variables; however, this framework does not permit conclusions regarding directionality. To examine directed relationships, we additionally implemented a Bayesian network representing variables within a directed acyclic graph and allowing the separation of direct predictive connections from indirect dependencies [40]. Network structure learning was performed using the hill-climbing (HC) procedure available in the bnlearn package. Model refinement involved iterative edge addition, deletion, and reversal, with improvements evaluated according to the Bayesian information criterion (BIC). In the construction of the Bayesian network, we employed the HC algorithm, allowing all possible edges between nodes. Although this approach allows a data-driven exploration of potential dependencies, it may also increase the risk of including spurious associations. Therefore, the resulting network structure should be interpreted with caution. The model was evaluated using the BIC to balance fit and complexity. To assess stability, bootstrap resampling with 1000 iterations was conducted, and edge thresholds were set according to strength-based criteria, including values such as 0.5 and 0.85. Two visualization methods were used: glasso for network structure and mgm for a detailed representation.

Directional dependencies were visualized using arrows to denote the orientation of probabilistic influence. Finally, network graphs were generated using the visualization strategy proposed by Sachs et al. (2005) [41], which applies a comparatively conservative shrinkage scheme, and that described by Scutari and Nagarajan (2013) [42]. Thus, two complementary Bayesian network representations were generated for interpretation.

## 3. Results

### 3.1 Descriptive Statistics and Exploratory Gender Comparisons

Node names and additional demographical information can be found in Table 1. Mean age at illness onset was 28.26 ± 9.95 years and was lower in males (27.02 ± 9.38 vs. 29.62 ± 10.46 years; *p *= 0.004). Illness duration, number of hospitalizations, current dosage of antipsychotic medicine, and length of current hospitalization did not differ by gender (all *p* > 0.05). Medication exposure duration was longer in males (17.15 ± 13.09 vs. 14.63 ± 11.49 years; *p *= 0.031).

**Table 1.** **Demographic information and gender differences**.

Variable (Unit)	Total (447)	Males (232)	Females (215)	*t*	*p*
Age (years)	49.09 ± 13.36	47.82 ± 13.75	50.47 ± 12.81	−2.100	0.036
Onset (years)	28.26 ± 9.95	27.02 ± 9.38	29.62 ± 10.46	−2.871	0.004
Course (years)	20.73 ± 12.71	20.73 ± 13.00	20.76 ± 12.76	−0.051	0.959
History (years)	4.12 ± 2.95	4.22 ± 3.24	4.07 ± 2.64	0.464	0.643
Equivalent (mg)	11.54 ± 7.11	11.10 ± 6.52	12.03 ± 7.70	−1.360	0.175
Drug Duration (years)	15.94 ± 12.40	17.15 ± 13.09	14.63 ± 11.49	2.160	0.031
Length of Current Hospitalization (years)	5.48 ± 6.88	5.49 ± 6.89	5.46 ± 6.88	0.050	0.963
PANSS (score)	53.87 ± 29.80	57.39 ± 28.14	50.08 ± 31.12	2.600	0.010
SSRS (score)	21.27 ± 6.60	21.26 ± 7.07	21.27 ± 6.06	−0.010	0.991
CD-RISC (score)	54.93 ± 17.07	55.14 ± 17.54	54.70 ± 16.59	0.270	0.785
ISMI (score)	59.41 ± 19.06	59.71 ± 18.97	59.08 ± 19.20	0.350	0.729
SQLS (score)	45.20 ± 22.26	40.55 ± 20.04	50.21 ± 23.45	−4.670	<0.001
CQSP (score)	146.44 ± 33.47	147.49 ± 31.95	145.30 ± 35.07	0.690	0.492
TEPS (score)	69.29 ± 17.72	69.75 ± 18.58	68.80 ± 16.77	0.560	0.574
TEPS-F1 (score)	14.79 ± 4.68	14.79 ± 4.91	14.79 ± 4.43	0.010	0.996
TEPS-F2 (score)	15.34 ± 4.81	15.17 ± 4.89	15.53 ± 4.73	−0.790	0.432
TEPS-F3 (score)	21.76 ± 6.08	21.53 ± 6.06	22.00 ± 6.11	−0.830	0.406
TEPS-F4 (score)	17.40 ± 6.13	18.25 ± 6.40	16.48 ± 5.69	3.100	0.002
SEI (score)	26.57 ± 11.04	24.16 ± 9.22	29.16 ± 12.21	−4.860	<0.001

Note: Age, current age of patients; Onset, age at first onset of schizophrenia; Course, duration of illness; History, number of previous hospitalizations; Equivalent, current medication dosage; Drug Duration, medication time; Length of Current Hospitalization, hospitalization duration; TEPS, Temporal Experience of Pleasure Scale; TEPS-F1, abstract anticipatory; TEPS-F2, contextual anticipatory; TEPS-F3, abstract consummatory; TEPS-F4, contextual consummatory; PANSS, Positive and Negative Syndrome Scale; SSRS, Social Support Rating Scale; CD-RISC, Connor–Davidson Resilience Scale; ISMI, Internalized Stigma of Mental Illness; SQLS, Schizophrenia Quality of Life Scale; CQSP, Coping Questionnaire for Schizophrenic Patients.

PANSS total scores were higher in males (57.39 ± 28.14 vs. 50.08 ± 31.12; *p* = 0.010). No significant gender differences were observed in SSRS, CD-RISC, ISMI, CQSP, or total TEPS scores and F1–F3 subscale scores (all* p* > 0.05).

Females reported higher SQLS scores (50.21 ± 23.45 vs. 40.55 ± 20.04; *p *< 0.001), whereas males scored higher on the contextual consummatory (TEPS-F4) subscale (*p* = 0.002). Females also showed higher SEI scores (29.16 ± 12.21 vs. 24.16 ± 9.22;* p *< 0.001). These clinical characteristics were reported to describe the sample and provide context for interpreting our network findings. However, they were not explicitly modeled as adjustment variables in primary network analyses and should therefore be considered as potential confounders when interpreting the observed associations. In addition, because the PANSS total score was used in the primary network analyses, heterogeneous relationships between specific symptom dimensions and psychosocial variables may have been obscured.

### 3.2 Regularized Graphical Network (Undirected)

**Fig. 1.**
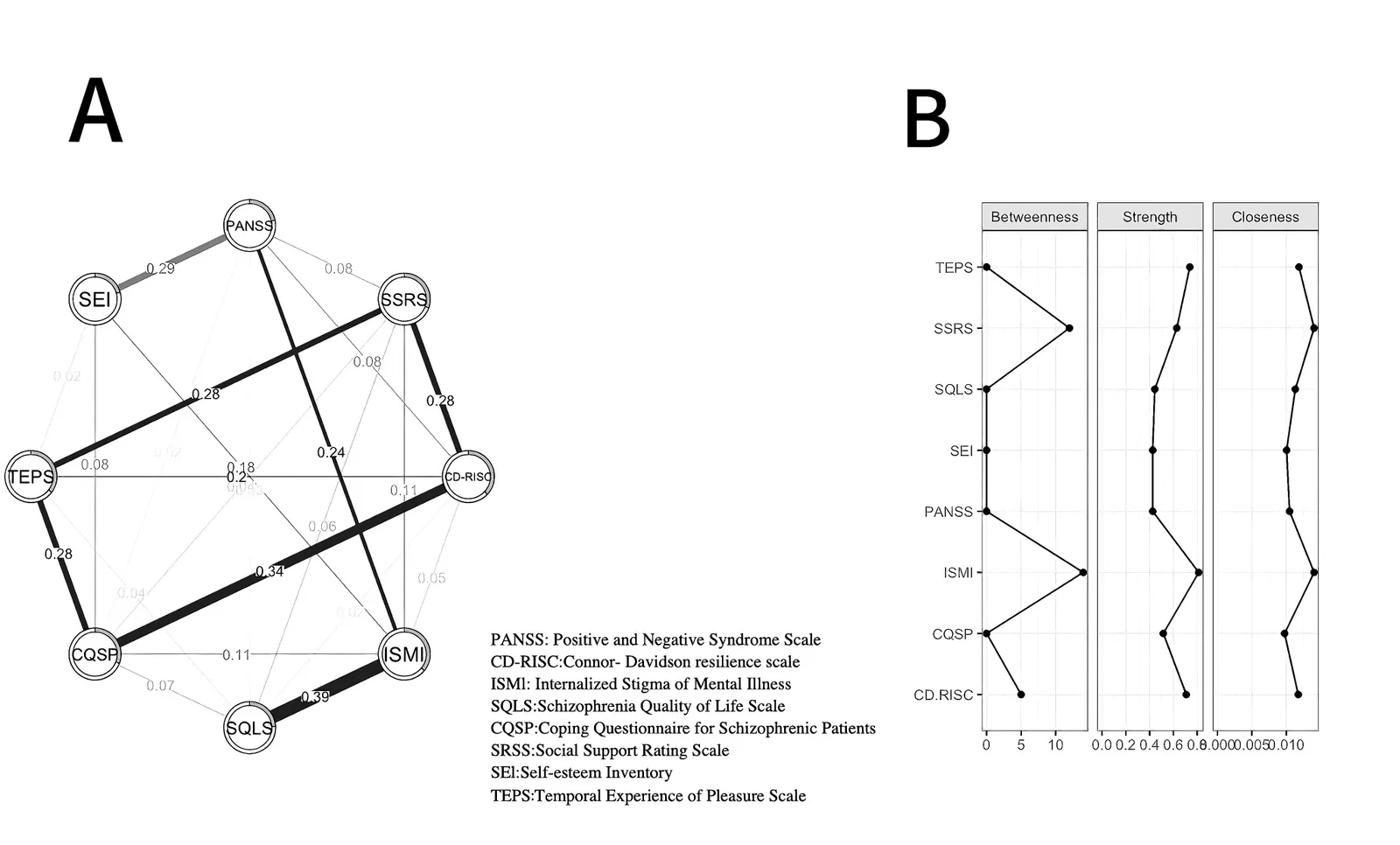
**Regularized partial correlation network and node centrality indices**. (A) Regularized partial correlation network estimated using the full dataset (*n* = 447). Each node corresponds to a subscale. Positive and negative relationships are shown in black and white, respectively. The surrounding black circle indicates node predictability, while thicker edges reflect stronger partial correlations, regardless of direction. (B) Strength refers to the total weighted connectivity of a node. Closeness captures how near a node is to all other nodes, and betweenness reflects the extent to which a node functions as a connector between different network regions.

A regularized undirected network was established to examine associations among symptom severity and psychosocial variables (Fig. 1A). Only edges with positive partial correlations of ≥0.20 and negative partial correlations of ≤−0.10 were reported.

The PANSS score showed a negative association with the SEI score (−0.29) and a positive association with the ISMI score (0.24). Among psychosocial variables, the strongest positive edge was observed between ISMI and SQLS scores (0.39). The CD-RISC score was positively correlated with CQSP (0.34) and perceived SSRS (0.28) scores. The TEPS score was positively connected with the overall CQSP score (0.28). Additional negative associations were observed between PANSS and CQSP scores (−0.18) and between CD-RISC and ISMI scores (−0.11). SQLS, ISMI, and CD-RISC scores showed relatively high predictability. Network accuracy and stability were supported by bootstrap analyses.

The nodes with the highest and second-highest betweenness centrality were ISMI and SSRS scores, respectively (Fig. 1B). The nodes with the highest, second-highest, and third-highest strength centrality were ISMI, TEPS, and CD-RISC scores, respectively. Regarding closeness centrality, ISMI and SSRS scores showed the highest value, followed by the CD-RISC score. Overall, psychosocial variables such as social support and internalized stigma served as key connectors in the network, with the ISMI score showing the highest observed centrality in the current sample. Network accuracy and stability were confirmed using bootstrapping tests (see **Supplementary Materials**).

**Fig. 2.**
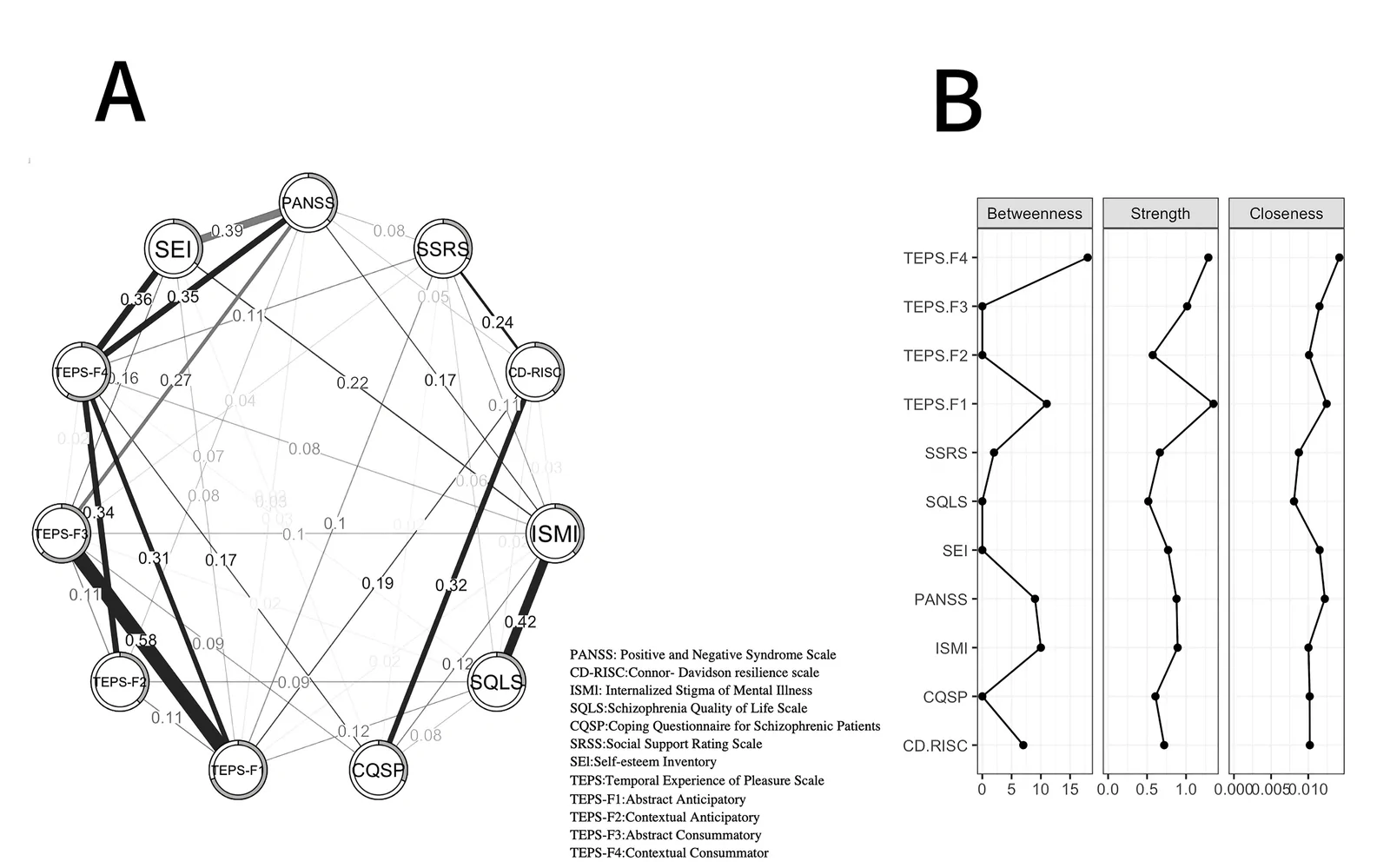
**Regularized partial correlation network and node centrality indices formed with TEPS nodes**. (A) Regularized partial correlation network estimated using the full dataset (*n* = 447). Each node corresponds to a subscale. Positive and negative relationships are shown in black and white, respectively. The surrounding blue circle indicates node predictability, while thicker edges reflect stronger partial correlations, regardless of direction. (B) Strength refers to the total weighted connectivity of a node. Closeness captures how near a node is to all other nodes, and betweenness reflects the extent to which a node functions as a connector between different network regions.

In subsequent analysis with TEPS subscale scores as individual nodes, strong internal coherence was shown for the TEPS score (Fig. 2A). The abstract anticipatory (TEPS-F1) score was strongly and positively correlated with the abstract consummatory (TEPS-F3) score (0.58), which represents the strongest association in the entire network. The TEPS-F1 score was also positively correlated with the TEPS-F4score (0.31). The ISMI score showed a strong positive correlation with the SQLS score (0.42). The SEI score was negatively correlated with PANSS (−0.39) and ISMI (−0.22) scores. The PANSS score was negatively correlated with the TEPS-F4 score (−0.27). The CQSP score was positively correlated with the CD-RISC score (0.32), and resilience was positively associated with the SSRS score (0.24).

The nodes with the highest, second-highest, and third-highest betweenness centrality were TEPS-F4, TEPS-F1, and ISMI scores, respectively (Fig. 2B). The nodes with the highest, second-highest, and third-highest strength centrality were TEPS-F1, TEPS-F4, and TEPS-F3 scores, respectively. The nodes with the highest, second-highest, and third-highest closeness centrality were TEPS-F4, TEPS-F1, and PANSS scores, respectively. These results suggest that specific anhedonia-related dimensions, particularly TEPS-F1 and TEPS-F4 scores, occupied prominent positions in the network estimated from the present sample. Accuracy and stability were confirmed using bootstrapping tests (see **Supplementary Materials**).

### 3.3 Bayesian Network (Directed)

**Fig. 3.**
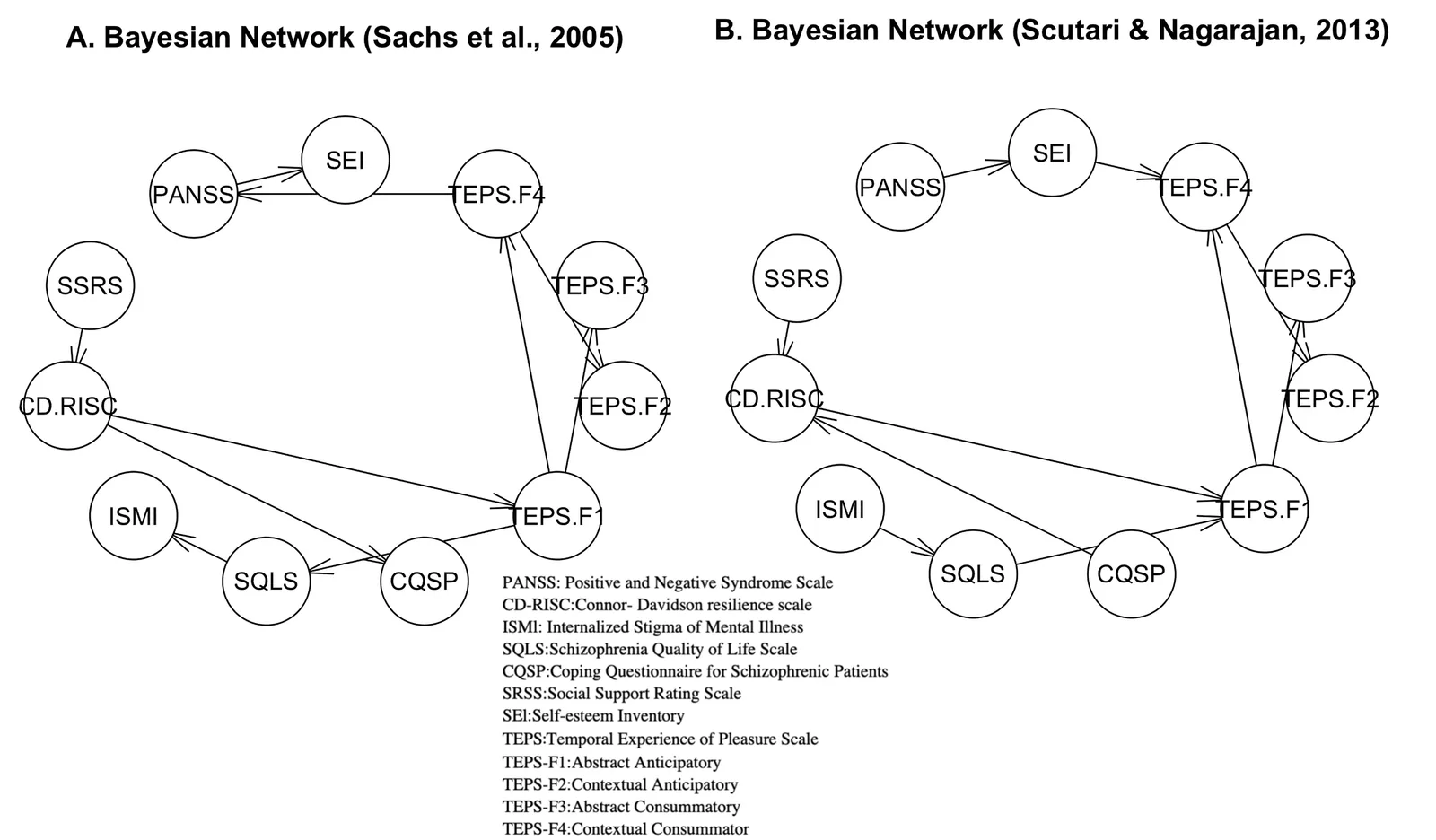
**Bayesian networks inferred from directed data (*n* = 447)**. Networks constructed using the approach proposed by (A) Sachs et al. (2005) [41] and (B) Scutari and Nagarajan (2013) [42]. Directed edges are shown with arrowheads indicating the inferred directionality. Line width reflects the probability associated with each predicted edge.

A directed acyclic graph was estimated using the 85% inclusion threshold described by Sachs et al. (2005) [41] to characterize the first Bayesian network (Fig. 3A, Ref. [41,42]). The resulting model delineated a set of conditional dependencies among symptom severity, psychosocial resources, and pleasure-related functioning. Within this structure, the TEPS-F1 score emerged as a downstream node receiving directed connections from the CD-RISC score. TEPS subdomain (F1–F4) scores were organized in a sequential configuration, consistent with an internally structured relationship among pleasure-related components. The SSRS score was linked to the CD-RISC score, which in turn connected to the CQSP or TEPS-F1 score. The TEPS-F1 score was further connected to TEPS-F3, TEPS-F4, and SQLS scores. The SQLS score was linked to the ISMI score. The TEPS-F4 score was connected to the contextual anticipatory (TEPS-F2) and PANSS scores, while the PANSS score to the SEI score.

The second Bayesian network, estimated using the method proposed by Scutari and Nagarajan (2013) [42], is presented in Fig. 3B. Although the overall topology of this network was comparable to that of the first model, it had a greater density of directed edges. The PANSS score was linked to the SEI score, which connected to the TEPS-F4 score and subsequently to the TEPS-F2 score. The SSRS score was linked to the CD-RISC score, which connected to the TEPS-F1 score, then to the TEPS-F4 score, and further to the TEPS-F2 score.

Furthermore, the ISMI score was linked to the SQLS score, which connected to the TEPS-F1 score and subsequently to the TEPS-F4 score. Importantly, associations involving CD-RISC and TEPS-F1 scores, as well as the pathway linking the SEI score to the TEPS-F4 score, were consistently retained across both network estimation approaches. Taken together, these findings suggest that several directed dependencies, particularly those involving CD-RISC-, TEPS-F1-, and SEI-related pathways, were retained across both network representations.

Although the Bayesian network revealed directional relationships among variables, these associations represent predictive priority rather than definitive causal effects. Causal inferences require further evidence from experimental designs or longitudinal studies, as observational data and network models cannot exclude the possibility of confounding variables or reverse causality.

To ensure network robustness, we conducted comprehensive stability analyses. **Supplementary Fig. 1** displays the expected influence of nodes across multiple psychological assessment scales. The case-dropping bootstrap test (**Supplementary Fig. 2**) showed stable strength centrality (Correlation Stability Coefficient [CS-C] = 0.75) across 90%–30% sampling proportions. This value exceeds the commonly recommended stability threshold of 0.50, supporting the reliability of the centrality rankings. Bootstrap confidence intervals (**Supplementary Figs. 3,4**) confirmed that ISMI-related edges (e.g., ISMI–SQLS and ISMI–SEI) had significantly consistent estimates with minimal sampling variability. These metrics, alongside correlation patterns (**Supplementary Fig. 5**) and bridge strength results (**Supplementary Fig. 6**), confirm the statistical reliability of our centrality rankings. Several Bayesian methods were also employed (**Supplementary Figs. 7–9**).

## 4. Discussion

Two network analytic approaches yielded graphs illustrating the complex interrelationships among schizophrenia-related symptoms, psychological resilience, internalized stigma, life quality, social support, coping strategies, self-esteem, and anticipatory/consummatory pleasure.

Mostly in line with our hypotheses, the PANSS score was negatively correlated with the SEI score and TEPS and TEPS subdimension scores, which aligns with clinical observations of more severe positive and negative symptoms being often associated with lower self-esteem and reduced hedonic capacity [43,44]. This network pattern agrees with previous findings of self-esteem being strongly interrelated with overall-, affective-, and negative-symptom severity in schizophrenia, indicating that more severe clinical symptoms are linked to poorer self-evaluative functioning [43]. Consistently, a meta-analysis showed that self-stigma is positively associated with psychotic symptom severity and strongly negatively correlated with self-esteem, reinforcing the relationship between clinical burden and diminished self-worth [21]. Furthermore, empirical evidence directly examining the relationship between self-esteem and clinical symptoms in schizophrenia further supports this robust association [45].

Positive edges linking the CQSP score to CD-RISC and ISMI scores were also uncovered. Specifically, coping strategies assessed using the CQSP score showed significant relationships with CD-RISC and ISMI scores, and positive coping strategies predicted lower self-stigma and improved life quality [20,46]. Changes in resilience have been shown to be significantly linked to those in self-stigma in patients with schizophrenia [47]. Additionally, the SSRS score demonstrated strong positive associations with CD-RISC and PANSS scores, which suggests that social support is closely linked to both resilience and symptom severity in patients with schizophrenia and agrees with prior findings of resilience mediating the relationship between negative symptoms and functional outcomes [48]. Moreover, the most pronounced interscale connection was observed between TEPS-F3 and CQSP scores, highlighting a critical relationship between consummatory pleasure and adaptive coping. This aligns with the ability of positive coping strategies to mitigate stress in caregivers and patients [49] and underscores the relevance of hedonic experience in schizophrenic populations [12].

The SQLS score showed a positive association with the ISMI score, which suggests that higher self-stigma is related to poorer perceived life quality and aligns with prior findings highlighting self-stigma as a critical psychosocial predictor of life satisfaction [11,20]. Conversely, the SQLS score exhibited a negative association with the TEPS-F1 score (anticipatory pleasure), which indicates that diminished capacity to anticipate pleasure might exacerbate reductions in life quality [50,51]. These relationships underscore the dual influence of negative affective experiences and internalized stigma on the subjective well-being of individuals with schizophrenia.

In the two Bayesian networks, SSRS and PANSS scores were positioned at the highest place, which suggests their directional associations. As per the network structures, the upstream scales (SSRS, PANSS, CQSP, and ISMI scores) had an impact on intermediate nodes (CD-RISC, SEI, and SQLS scores), and ultimately on TEPS subdimensions. Simultaneously, some intermediate nodes also influenced each other. For example, the CD-RISC score affected TEPS-F1 and CQSP scores, while the TEPS-F1 score influenced TEPS-F3 and TEPS-F4 scores. Thus, psychological resilience might have a significant impact on the subdimensions of anhedonia and coping strategies of individuals with schizophrenia.

The TEPS-F1 score acted as a key connecting node between upstream scales and downstream TEPS subdimensions, thus potentially being a critical target for interventions aimed at modifying the pathways to other TEPS factors. The TEPS score was reported to exhibit a stable multidimensional structure in healthy and clinical populations, with anticipatory pleasure serving as a foundational dimension of overall pleasure experience and exerting a particularly significant regulatory effect on subsequent experiences [7,12]. The central role of the TEPS-F1 score might be closely associated with individual psychological resilience and coping ability. Previous research indicated that psychological resilience quantified using the CD-RISC score predicts an individual’s level of pleasure experience in various contexts and showed that the mediating role of TEPS-F1 might make it a critical pathway through which upstream psychological resources influence downstream pleasure experiences [52]. Collectively, the demonstrated stability and multidimensional organization of the TEPS support the interpretation of the central role of TEPS-F1 observed in the present network analysis [53,54].

Consistent with prior research employing Bayesian network approaches, our findings indicate that the effects of upstream measures, such as PANSS and SSRS scores, on TEPS subdimension scores are predominantly mediated by intermediate psychological factors (SEI, CD-RISC, and SQLS scores) rather than exert direct influences [55,56,57]. Specifically, the identified pathway is that social support affects anhedonia by influencing psychological resilience, which suggests the presence of a shared psychosocial mechanism in which social support enhances psychological resilience and thus shapes hedonic and anticipatory pleasure experiences. This pattern aligns with previous evidence of resilience serving as a critical mediator linking social and emotional factors to mental health outcomes [58] and psychosocial risk and protective factors often operating indirectly through sequential mediators rather than via direct effects [59,60]. Collectively, these findings provide a plausible explanation for the minimal direct influence of upstream scales on TEPS subdimension scores observed in our study, highlighting the central role of intermediate psychological constructs in the etiology of pleasure-related deficits.

Another important finding is the structural discrepancy between the two networks. Despite both models focusing on TEPS subdimension scores, Model A [41] positioned the TEPS-F2 score as the most downstream node, while Model B (Scutari & Nagarajan, 2013) [42] positioned the TEPS-F2 score as a consequence of the TEPS-F4 score. However, both models converged on the central role of the TEPS-F1 score in linking upstream resilience and cognitive factors to downstream anhedonia traits. Previous research indicated that the relative positions and interrelations of TEPS subdimension scores vary across samples [12], with measurable variance observed across gender but minor structural differences observed between anticipatory and consummatory components [53]. Previous findings on anhedonia networks and TEPS structure provide support for our findings [61,62]. These findings align with our models, supporting the observed variability in TEPS-F2 score positioning and the integrative role of the TEPS-F1 score, which bridges upstream psychological factors and pleasure-related outcomes.

The present findings should also be interpreted in light of potentially relevant clinical characteristics. Although the sample was restricted to clinically stable inpatients receiving stable antipsychotic treatment for at least 1 month, variability in illness duration, antipsychotic dose equivalent, medication exposure duration, and overall symptom severity may still have influenced hedonic functioning and psychosocial variables. For example, chronicity, cumulative medication exposure, and greater psychopathology burden may each be associated with lower pleasure experience, poorer self-esteem, and greater internalized stigma. As these clinical variables were not explicitly modeled as covariates in primary network analyses, some of the observed associations may partly reflect residual confounding. Future studies should examine whether the network structure remains stable after incorporating medication- and illness-related clinical factors more directly.

Taken together, the results obtained using Bayesian networks suggest that the TEPS-F1 score and its upstream drivers (e.g., CD-RISC and CQSP scores) might be considered primary targets for intervention in populations with anhedonia-related symptoms. Such interventions could aim to modify the underlying cognitive and resilience factors and thus improve motivational and emotional outcomes in these populations. In clinical practice, patients with schizophrenia and severe anhedonia showed improvement in anticipatory pleasure and daily activity after 10–25 h of cognitive-sensory intervention [63]. Guided thinking of positive future events enhances anticipatory pleasure, vividness, and mental imagery, supports behavioral intentions, and has clinical relevance for mood and schizophrenia-spectrum disorders [62]. In Guangzhou (China), Li et al. [54] conducted a randomized controlled trial (RCT) using a community-based approach to reduce internalized stigma and discrimination, revealing significant reductions in anticipated discrimination and an increase in overcoming stigma among schizophrenia patients. Notably, the “Against Stigma Program” significantly reduced self-stigma and increased self-esteem, achieving stronger effects compared with the control group immediately after the intervention and at a 1-month follow-up [64]. These findings suggest that interventions targeting anticipatory pleasure through positive future thinking and stigma reduction might significantly improve affective symptoms, daily functioning, and self-esteem in schizophrenia patients, serving as effective adjuncts to pharmacological treatments and enhancing overall psychosocial outcomes.

The ISMI score emerged as the most influential variable in the overall scale, which reflects its pivotal position. Across clinical and nonclinical populations, central nodes have repeatedly been shown to drive network dynamics, which supports the relevance of ISMI score centrality in shaping overall symptom patterns [60,65]. Subscale-specific analyses revealed domain-dependent centrality, as the TEPS-F4 score dominated the anhedonia network, consistent with prior reports linking motivation- and pleasure-related variables to central roles within social and affective symptom networks [61,66].

### Limitations

Although Bayesian network analysis highlighted potential relationships among variables, it cannot lead to causal claims. Another limitation concerns diagnostic ascertainment and the exclusion of secondary or organic causes of psychosis. Although all participants were diagnosed by experienced psychiatrists and clinically screened using routine inpatient evaluation and medical record reviews, the present study did not prospectively collect a standardized research battery of ancillary examinations, such as neuroimaging, electroencephalography, lumbar puncture, toxicology screening, or comprehensive laboratory testing, for uniform reporting across all participants. Therefore, we were unable to systematically classify patients into idiopathic versus organic/secondary psychosis subgroups or conduct subgroup analyses on this basis. As a result, residual diagnostic heterogeneity cannot be fully excluded, and the findings should be interpreted with caution, particularly when considering the specificity of the observed associations to primary schizophrenia. Additionally, the sample was drawn from a single psychiatric inpatient facility in Beijing, which may limit the generalizability of the findings to broader groups. The requirement of at least a junior high school education may have introduced selection bias, as individuals with lower literacy or educational attainment were not included. This may further limit the generalizability of our findings, particularly to patients with poorer literacy, lower educational backgrounds, or greater cognitive and functional disadvantages. Therefore, the present findings may not be fully generalizable to individuals with lower educational attainment, who may differ in cognitive, clinical, and psychosocial characteristics. Interrater reliability for the PANSS score was estimated based on only 3 parallel interviews; therefore, the ICC estimates were derived from a very small sample and may have limited stability and precision, warranting cautious interpretation. Although routine calibration, regular rater meetings, case conference review, and adjudication procedures were used to support rating consistency, this small subsample should still be considered a methodological limitation. Lastly, the use of self-report measures may have introduced social desirability bias, especially in institutionalized environments. Future studies could benefit from integrating ecological momentary assessment, behavioral tasks, or informant reports to complement subjective data and reduce potential biases, thereby enhancing the robustness and validity of findings.

## 5. Conclusions

In the examined cross-sectional sample of patients with schizophrenia, internalized stigma occupied a prominent position in the overall psychosocial network, whereas abstract anticipatory and contextual consummatory pleasure showed prominent positions when anhedonia-related subdimensions were modeled separately. Across two Bayesian network representations, recurrent directional association patterns linked (i) social support and resilience-related variables with pleasure-related subdimensions and (ii) overall psychopathology/self-esteem-related variables with hedonic functioning. These findings support the relevance of self-stigma and anhedonia-related domains as important candidates for future longitudinal and intervention-focused research in schizophrenia. However, given the cross-sectional design and observational nature of the data, the present findings should be interpreted as associative patterns rather than evidence of causal relationships.

## Data Availability

The datasets generated and analyzed during the current study are available from the corresponding author on reasonable request.
