# Supplementary material for: Disentangling Anhedonia and Self-Stigma in Schizophrenia Using Bayesian Network Analysis
Source: Alpha Psychiatry. 2026 Jul 28;27(4):50731. doi: 10.31083/AP50731 (PMC13540050; doi:10.31083/AP50731)
Supplement: Supplementary file 1 [file 2757-8038-27-4-50731-s1.zip › Supplementary Figures.docx]

**Supplementary Fig. 1. Expected Influence**

Expected influence (EI) of nodes in the EBICglasso network (γ = 0.25). EI reflects overall connectivity, accounting for both positive and negative edge weights.

**Supplementary Fig. 2. Case-dropping Bootstrap Analysis of Centrality Stability**

This figure shows the results of the case-dropping bootstrap procedure used to assess the stability of centrality indices. Stability was quantified using the correlation stability (CS) coefficient, with higher values indicating more robust centrality estimates.

**Supplementary Fig. 3. Bootstrap Confidence Intervals for Edge Weights**

This figure displays nonparametric bootstrap confidence intervals for edge weights in the EBICglasso network, reflecting the accuracy of estimated partial correlations.

**Supplementary Fig. 4. Bootstrap Edge Weight Accuracy**

Bootstrap results for edge weights in the EBIC glasso network are shown. Black points represent bootstrap means, red points indicate sample estimates, and error bars depict 95% confidence intervals, reflecting the stability and precision of the edge weight estimates.

**Supplementary Fig. 5. Pearson Correlation Matrix**

This figure shows the Pearson correlation matrix of all variables. These zero-order correlations are presented for descriptive purposes and differ from the partial correlations estimated in the network models.

**Supplementary Fig. 6. Bridge Strength Centrality**

This figure displays bridge strength centrality indices, quantifying the extent to which each node connects different communities within the undirected network.

**Supplementary Fig. 7. Bayesian Network (Threshold = 0.85)**

This figure shows the averaged Bayesian network derived from 1000 bootstrap samples using the hill-climbing (hc) algorithm. Only edges with arc strength ≥ 0.85 are retained, representing highly stable relationships.

**Supplementary Fig. 8. Bayesian Network (Default Threshold)**

This figure presents the averaged Bayesian network using the default arc strength threshold. Compared to stricter thresholds, more edges are retained, including moderately stable relationships.

**Supplementary Fig. 9. Bayesian Network (Threshold = 0.5)**

This figure shows the averaged Bayesian network with a lower threshold (0.5), resulting in a denser structure that includes less stable edges.
